# Swords and shields: the war between *Candidatus* Liberibacter asiaticus and citrus

**DOI:** 10.3389/fpls.2024.1518880

**Published:** 2025-01-07

**Authors:** Yanan Hu, Nannan Lu, Kaiqiang Bao, Shuting Liu, Ruimin Li, Guiyan Huang

**Affiliations:** ^1^ College of Life Sciences, Gannan Normal University, Ganzhou, China; ^2^ National Navel Orange Engineering Research Center, Gannan Normal University, Ganzhou, China; ^3^ Jiangxi Provincial Key Laboratory of Pest and Disease Control of Featured Horticultural Plants, Gannan Normal University, Ganzhou, China

**Keywords:** Citrus sinensis, *Candidatus* Liberibacter asiaticus, Sec-dependent effectors, interaction, virulence

## Abstract

Citrus Huanglongbing (HLB) represents a significant threat to the citrus industry, mainly caused by the phloem-limited bacterium *Candidatus* Liberibacter asiaticus (*C*Las). In this review, we summarize recent advances in understanding the relationship between citrus and *C*Las, particularly examining the functions of Sec-dependent effectors (SDEs) and non-classically secreted proteins (ncSPs) in virulence, as well as their targeted interactions with citrus. We further investigate the impact of SDEs on various physiological processes, including systemic acquired resistance (SAR), reactive oxygen species (ROS) accumulation, vesicle trafficking, callose deposition, cell death, autophagy, chlorosis and flowering. Additionally, we focus on the functional research on specific disease-resistant genes in citrus and the molecular mechanisms underlying disease resistance. Finally, we discuss the existing gaps and unresolved questions regarding citrus-*C*Las interactions, proposing potential solutions to facilitate the development of HLB-resistant citrus varieties.

## Introduction

Citrus Huanglongbing (HLB), caused by the phloem-restricted bacterium *Candidatus* Liberibacter asiaticus (*C*Las), americanus (*C*Lam), and africanus (*C*Laf), is one of the most devastating diseases affecting the citrus industry (Bové, 2006; Wang, 2019; Sivager et al., 2021). Currently, *C*Las is most widely spread in citrus production areas in Asia and America (Zhou, 2020; Alquézar et al., 2021b). The disease is primarily transmitted in the field by the Asian citrus psyllid (*Diaphorina citri* Kuwayama). *C*Las proliferates within the psyllid and is subsequently transmitted to the phloem sieve elements of host plants through the insect’s saliva during feeding on young shoots (Hall et al., 2013; Alquézar et al., 2021a). *C*Las infects various citrus tissues, including stems, leaves, fruits, and roots, leading to phloem blockages that result in stunted growth, reduced fruit size, elevated juice acidity, and potentially tree mortality (Gottwald, 2010; Ma et al., 2022).

The genome of the *C*Las is approximately 1.2 ~ 1.3 Mb and lacks type III and type IV secretion systems, while containing a complete type I secretion system and the general secretory (Sec) pathway (Duan et al., 2009; Zheng et al., 2024). The Sec pathway is essential for the transmembrane transport of bacterial proteins, and the Sec-dependent effectors (SDEs) are important virulence factors of phloem-colonizing bacteria that cause plant diseases (Sugio et al., 2011; Tomkins et al., 2018). Thus, elucidating the biological functions of SDEs in the *C*Las infection process in citrus could provide valuable insights into the pathogenic mechanisms utilized by *C*Las.

To comprehensively elucidate the biological interactions between *C*Las and citrus, as well as to deepen our understanding of *C*Las pathogenic mechanisms and citrus immune responses, this review summarizes recent research on SDEs and non-classically secreted proteins (ncSPs) in *C*Las. Additionally, this review also emphasizes genes validated *in vivo* that contribute to enhancing citrus resistance to HLB.

## *C*Las SDEs and ncSPs as pathogen weapons against citrus

To overcome the host’s defenses against bacterial proliferation, bacteria often deploy effectors to disrupt the host’s immune responses, thereby reducing the host’s resistance to infection (Shan et al., 2008; Wang et al., 2022). In *C*Las, there are 86 proteins that have been experimentally confirmed to have signal peptides (Prasad et al., 2016). Analyzing the biological processes that SDEs are involved in within plant cells can provide insights into the pathogenic mechanisms of *C*Las. It is observed that various SDEs are involved in the inhibition of specific processes in plant cells. When SDE15 (CLIBASIA_04025) interacts with ACCELERATED CELL DEATH 2 (ACD2), it has the effect of suppressing hypersensitive response (HR) cell death in plants (Pang et al., 2020). AGH17488 (a SDE in *C*Las strain gxpsy, its homologous protein in *C*Las strain psy62 is CLIBASIA_05590) is able to target and promote the enzyme activity of ascorbate peroxidase 6 (APX6) in citrus, ultimately leading to the inhibition of ROS accumulation (Du et al., 2023). Moreover, *C*Las0185 (CLIBASIA_00185) interacts with methionine sulphoxide reductase B1 (CsMsrB1) and boosts the enzyme activity of ascorbate peroxidase 1 (APX1) in citrus, resulting in a reduction of H_2_O_2_ content (Zhang et al., 2024). It is worth noting that SDE4310 (CLIBASIA_04310), SDE4435 (CLIBASIA_04435), and SDE4955 (CLIBASIA_04955), which are able to inhibit cell death and ROS accumulation, are discovered to interact with *Arabidopsis thaliana* CAT3 and GAPA (Li et al., 2024). Furthermore, m3875 (CLIBASIA_03875), m4405 (CLIBASIA_04405), and SECP8 (CLIBASIA_05330) are identified as suppressors of ROS accumulation (Zhang et al., 2019, 2020; Shen et al., 2022). Specifically, m4405, also known as SDE4405, is referred to the same SDE in different literatures (Zhang et al., 2020; Shi et al., 2023a). In addition, SDE3 (CLIBASIA_00420) interacts with citrus cytosolic glyceraldehyde-3-phosphate dehydrogenases (CsGAPCs) causing impairment of autophagy in citrus, consequently diminishing plant immunity (Shi et al., 2023b). SDE19 (CLIBASIA_05320) interacts with Sec12, causing disruption to vesicle trafficking and callose deposition in plants (Huang et al., 2024a). Furthermore, the overexpression of *Ca*LasSDE115 (CLIBASIA_05115) impedes the citrus systemic acquired resistance (SAR) response and boosts the early establishment of *C*Las infection (Du et al., 2022).

However, some SDEs can greatly stimulate the host’s response. For instance, SDE1 (CLIBASIA_05315) has been shown to induce reactive oxygen species (ROS) accumulation, cell death, and chlorosis in plants (Clark et al., 2018; Pitino et al., 2018; Clark et al., 2020; Zhou et al., 2020). Additionally, studies have proven that SDEs like *Ca*LasSDE460 (CLIBASIA_00460) can cause chlorosis and cell death (Liu et al., 2019; Wang et al., 2023), while FlgI (CLIBASIA_01305) can trigger callose deposition and cell death (Zuo et al., 2024). Moreover, m3915 (CLIBASIA_03915) and m4250 (CLIBASIA_04250) have been shown to induce cell death (Li et al., 2020), and *C*Las4425 (CLIBASIA_04425) can also result in cell death and the accumulation of ROS (Zhang et al., 2023). SDE4405 (CLIBASIA_04405) has been found to interact with ATG8-family proteins (ATG8s) which leads to the stimulation of autophagy in plants (Shi et al., 2023a). Furthermore, it is interesting to note that SDE1 interacts with various citrus papain-like cysteine proteases (PLCPs) and the DEAD-box RNA helicase (DDX3) (Clark et al., 2018; Zhou et al., 2020). Since PLCPs and DEAD-box RNA helicase have been demonstrated to play a role in defense mechanisms against pathogen invasion (Li et al., 2008; Misas-Villamil et al., 2016), it can be inferred that SDE1 weakens the immune response in citrus by interacting with PLCPs and DEAD-box RNA helicase, thereby promoting *C*Las infection. Consequently, the query of whether these SDEs are utilized as weapons by *C*Las to counteract the host’s defense system in the infection process remains disputed and calls for additional research.

Among ncSPs, SC2_gp095 (No annotated homologous proteins were identified in the *C*Las strain psy62 according to a BLASTp search conducted on October 5, 2024.), a nonclassical secreted peroxidase of *C*Las, is capable of reducing the accumulation of ROS, thereby suppressing HLB symptoms (Jain et al., 2015). LasBCP (CLIBASIA_00445), a peroxiredoxin secreted by *C*Las, has the ability to suppress the SAR response and inhibit callose deposition in plants (Jain et al., 2018, 2019, 2021). Furthermore, ncSPs LasRNHI (CLIBASIA_03435) suppresses plant flowering by interacting with a citrus B-box zinc finger protein CsBBX28 to inhibit CsBBX28’s regulation of FLOWERING LOCUS T expression (Du et al., 2024).

**Figure 1:**
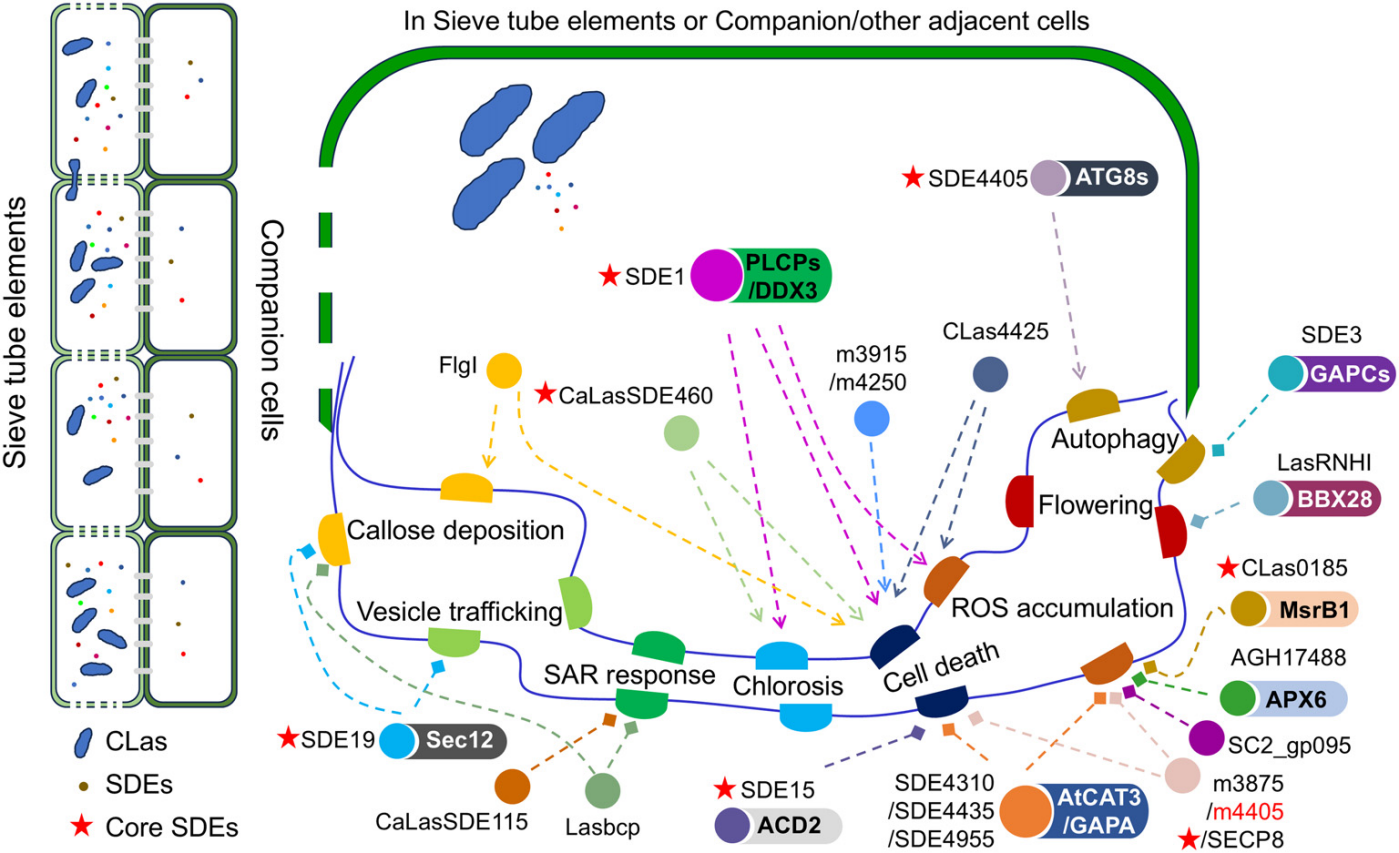
The interactions between *Candidatus* Liberibacter asiaticus (*C*Las) and citrus. The schematic diagram illustrates the virulence functions of Sec-dependent effectors (SDEs) and non-classically secreted proteins (ncSPs), along with their potential targets in citrus-*C*Las interactions. The dashed line ending with an arrow indicates activation, while the end marked with a diamond signifies suppression.

In brief, *C*Las uses SDEs and ncSPs as pathogen weapons to disrupt the regular functioning of citrus cells, suppress plant immune responses, and advance the infection process of *C*Las (**Figure 1**). Therefore, how can citrus combat *C*Las infection?

## Restricted defensive shields of citrus in response to *C*Las infection

The nonexpressor of pathogenesis-related genes 1 (NPR1) is a crucial activator in salicylic acid (SA)-mediated immune responses, exhibiting diverse roles in plant resistance to various pathogens (Zavaliev and Dong, 2024). Overexpression of *A. thaliana NPR1* (*AtNPR1*) significantly improved citrus resistance to *C*Las infection, likely by activating the citrus SA signaling pathway, which elevated the plant’s immune response (Qiu et al., 2020). Subsequently, overexpression of *CiNPR4*, an *NPR1*-like gene from *Citrus paradisi*, and *CsNPR1* from *C. sinensis* indicated that the transgenic lines exhibited enhanced resistance to HLB (Peng et al., 2021; Wu et al., 2021). Given that activation of the SA signaling pathway can bolster citrus resistance to HLB, the overexpression of SA methyltransferase (*CsSAMT1*) in citrus elevated levels of SA and methyl salicylate (MeSA), thereby enhancing resistance to *C*Las infection (Zou et al., 2021). Notably, the introduction of transgenic SA binding protein 2 (NtSABP2) from tobacco, which plays a role in systemic acquired resistance (SAR), also markedly improved citrus resistance to HLB (Soares et al., 2022).

Antimicrobial peptides (AMPs) are essential components of the plant immune response against bacterial infections (Campos et al., 2018). Recently, an exciting study has found that stable antimicrobial peptides (SAMPs) derived from *Microcitrus australiasica* Australian finger lime (MaSAMP) can strongly inhibit the proliferation of *C*Las (Huang et al., 2021). Moreover, A chimeric peptide, UGK17, has demonstrated bactericidal activities against *C*Las in citrus (Basu et al., 2022; Choi et al., 2023). These findings suggest that AMPs are an effective strategy for the prevention and control of HLB. Additionally, the overexpression of the endolysin gene *LasLYS2* (*CLIBASIA_04800*) in citrus provides significant dual resistance to both HLB and citrus canker, effectively preventing the colonization of *C*Las in transgenic plants (Xu et al., 2023).

In short, despite the fact that there are constrained resistance mechanisms to HLB, current research findings offer promising applications for the citrus industry. However, this approach faces considerable challenges and requires substantial effort.

## Discussion

The researchers have found that SDEs have an impact on various plant biological processes, including callose deposition, vesicle trafficking, SAR response, chlorosis, cell death, ROS accumulation, flowering, and autophagy (Clark et al., 2020; Pang et al., 2020; Du et al., 2023; Shi et al., 2023a, 2023; Du et al., 2024; Huang et al., 2024a; Zhang et al., 2024). It is interesting to note that certain SDEs can either promote or suppress ROS accumulation to facilitate *C*Las infection. For instance, SDE1 and *C*Las4425 can induce ROS accumulation, while *C*Las0185, AGH17488, m3875, m4405, SECP8, SDE4310, SDE4435, and SDE4955 can suppress it. The questions arise: why does both the induction and suppression of these biological processes benefit *C*Las infection in plants? Is *C*Las simultaneously regulating these processes during infection, or does it continuously change its strategies to alter the plant cell environment for its own survival throughout the infection process? Challenging work includes identifying which specific SDEs are critical for *C*Las infection and determining whether these SDEs operate independently or synergistically. If a synergistic interaction occurs, what regulatory mechanisms govern their interplay?

Although there are studies providing evidence that HLB is a pathogen immune-mediated disease (Ma et al., 2022), there are still many unanswered questions. For example, in the citrus-*C*Las interaction process, *C*Las induces or inhibits ROS accumulation through multiple SDEs or ncSPs (Jain et al., 2015; Clark et al., 2020; Du et al., 2023; Zhang et al., 2023; Li et al., 2024; Zhang et al., 2024). Additionally, in *C*Las infection samples, the expression trend of ROS metabolism-related genes is not completely reprogrammed. Out of the 91 ROS metabolism-related genes, 30 showed significant differential expression, with 16 being up-regulated and 14 down-regulated (Huang et al., 2024b). So, how does citrus trigger an ROS burst if it is not a result of *C*Las manipulating ROS accumulation during infection?

Fortunately, new technologies offer hope and illumination in tackling these scientific challenges. Despite the inability to culture *C*Las *in vitro* (Wang, 2019), spatial single-cell transcriptomics (Giacomello, 2021) can provide valuable insights into the *C*Las infection process in the citrus phloem tissues and the response of citrus to *C*Las infection. This can potentially address numerous unanswered questions. Additionally, due to the low abundance of *C*Las transcripts in citrus tissues, conventional sequencing methods may have limitations in obtaining sequencing reads (De Francesco et al., 2022). Higher-throughput sequencing instruments or methods, such as the NovaSeq X sequence platform and Data-independent acquisition (DIA) proteomics, can be utilized. Moreover, there have been 27 core SDEs identified in *C*Las (Thapa et al., 2020), but only 7 of them have been analyzed for their functions so far (**Figure 1**). The functions of most core SDEs remain unclear, so yeast two-hybrid and immunoprecipitation-mass spectrometry techniques can be used to identify targets of core SDEs in citrus, in order to obtain potential candidate susceptibility genes. Subsequently, by utilizing transgene-free CRISPR/Cas9 or Cas12a/crRNA technology to knock out these genes that interact with *C*Las SDEs in citrus (Wang, 2019; Alquézar et al., 2022; Su et al., 2023), it can be determined if disrupting the interaction can enhance citrus resistance to HLB and generate HLB-resistant citrus lines. Furthermore, it was observed that a full resistance to CLas was manifested in citrus relatives, such as *Eremocitrus glauca*, *Microcitrus warburgiana*, *M. papuana*, and *M. australis*, along with hybrids either among them or between them and Citrus (Alves et al., 2021, 2022). These germplasm resources offer a substantial genetic foundation for discerning citrus resistance to *C*Las infection and further bolster research pertaining to interactions between plants and phloem-invading pathogens. A prior study indicated that the overexpression of *AtNRP1* can boost citrus resistance to *C*Las (Qiu et al., 2020), thus reinforcing our conviction that resistance genes sourced from these citrus relatives will substantially contribute to enhancing citrus resistance to *C*Las.

In conclusion, the pathogenic mechanism of *C*Las remains unclear, and many mysteries surrounding citrus-*C*Las interaction still need to be unraveled. However, with the ongoing accumulation of research findings and the development of new experimental methods, we remain optimistic that the eradication of HLB in the citrus industry is within reach.
